# Novel research and future prospects of artificial intelligence in cancer diagnosis and treatment

**DOI:** 10.1186/s13045-023-01514-5

**Published:** 2023-11-27

**Authors:** Chaoyi Zhang, Jin Xu, Rong Tang, Jianhui Yang, Wei Wang, Xianjun Yu, Si Shi

**Affiliations:** 1 Department of Pancreatic Surgery, Fudan University Shanghai Cancer Center, No. 270 Dong’An Road, Shanghai, 200032 People’s Republic of China; 2 Department of Oncology, Shanghai Medical College, Fudan University, Shanghai, 200032 People’s Republic of China; 3 Shanghai Pancreatic Cancer Institute, No. 399 Lingling Road, Shanghai, 200032 People’s Republic of China; 4 Pancreatic Cancer Institute, Fudan University, Shanghai, 200032 People’s Republic of China

## Abstract

Research into the potential benefits of artificial intelligence for comprehending the intricate biology of cancer has grown as a result of the widespread use of deep learning and machine learning in the healthcare sector and the availability of highly specialized cancer datasets. Here, we review new artificial intelligence approaches and how they are being used in oncology. We describe how artificial intelligence might be used in the detection, prognosis, and administration of cancer treatments and introduce the use of the latest large language models such as ChatGPT in oncology clinics. We highlight artificial intelligence applications for omics data types, and we offer perspectives on how the various data types might be combined to create decision-support tools. We also evaluate the present constraints and challenges to applying artificial intelligence in precision oncology. Finally, we discuss how current challenges may be surmounted to make artificial intelligence useful in clinical settings in the future.

## Introduction

In the upcoming decades, it is anticipated that cancer would surpass other illnesses as one of the main global causes of morbidity and mortality [1]. A recent study from The Lancet [2] demonstrated that for many low-income and middle-income nations, noncommunicable diseases (NCDs) pose an ever-greater health threat, with cancer becoming an NCD of greater importance. Therefore, it is imperative to focus on cancer treatment, enhance the rate of early detection and cure, and boost cancer screening.

Due to technical advancements in statistics and computer software, computer professionals, and health scientists may now collaborate closely to improve prognoses. As a result of the adoption of artificial intelligence (AI) strategies, researchers have increasingly concentrated on creating models using AI algorithms to detect and diagnose cancer. AI is the process of teaching a computer to mimic human intelligence by showing it how to study, evaluate, comprehend, deduce, interact, and make decisions [3]. Tremendous success has been achieved with AI in the last ten years in the fields of speech synthesis, natural language processing, and computer vision. This review focuses on the latest AI techniques for tumor diagnosis, treatment, and prognosis. We highlight artificial intelligence applications for omics data types, and we offer perspectives on how the various data types might be combined to create decision-support tools and discuss how current challenges may be surmounted to make artificial intelligence useful in clinical settings in the future.

We searched three databases from their creation until November 10, 2023: MEDLINE (PubMed), CENTRAL (Cochrane Central Register of Controlled Trials), and Embase to assess the published literature pertaining to the application of artificial intelligence in cancer. Due to the rapid pace of AI updates, we have focused on the last two years of relevant research. The following keywords were used in this scoping review: (neoplasms OR cancer) AND (artificial intelligence OR deep learning OR machine learning). With a focus on the application and usage of artificial intelligence in cancer treatment, we incorporated a total of 254 publications in the construction of this narrative review, including pertinent prospective, retrospective, and review studies.

## Specific meaning of artificial intelligence

**Fig. 1:**
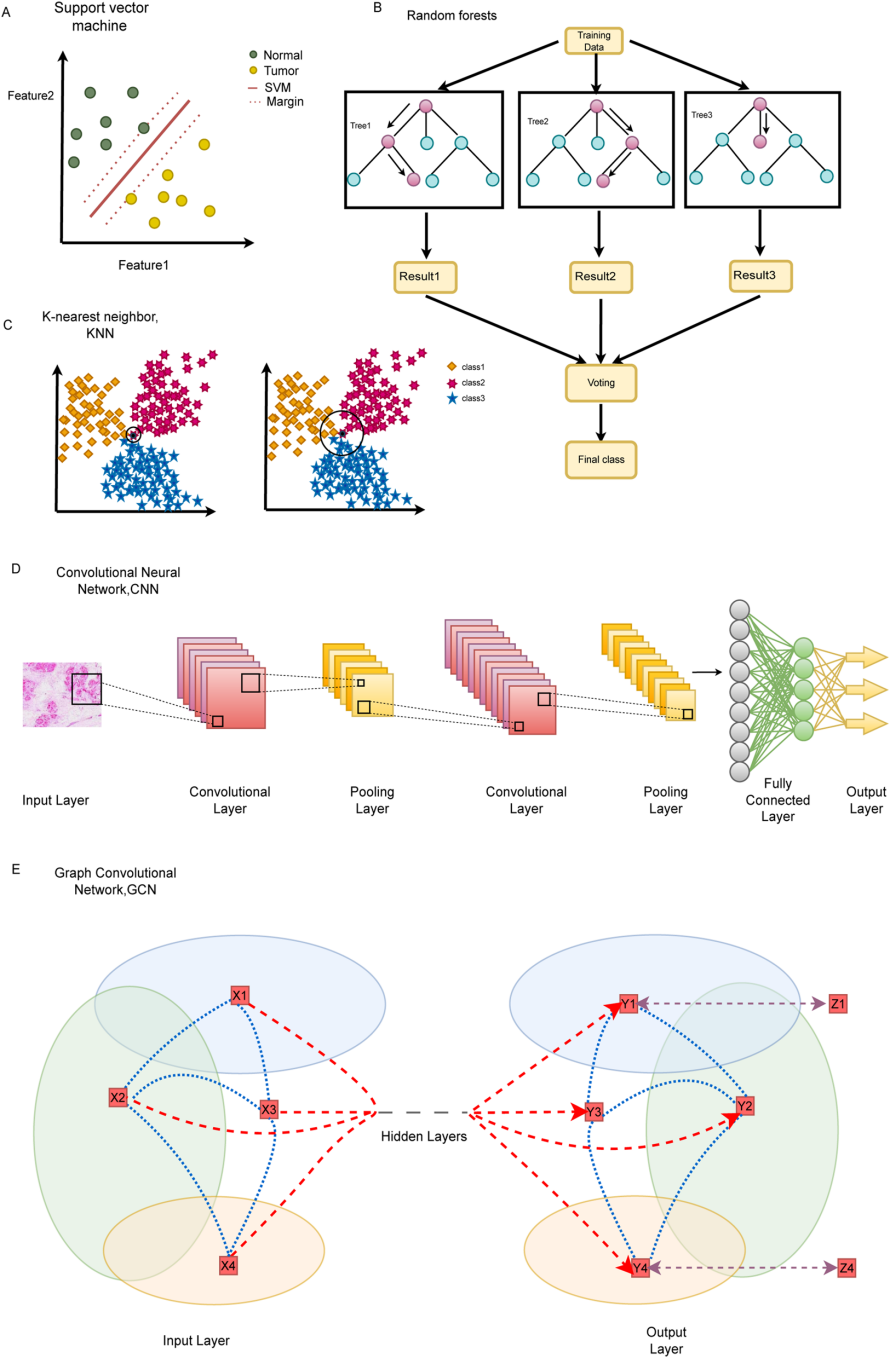
Network structure of DL. **a** A model of an SVM; **b** A model of a random forest that is composed of several decision trees; **c** KNN characterized by the fact that it is composed of many random features rather than a linear feature; **d** Components of CNNs [6]; and **e** Components of graphical CNNs

AI is an area of computer technology comprising numerous techniques and subfields aimed at performing activities that could previously be completed only by humans. To enhance the interpretation of medical data relevant to medical administration, diagnostics, and predictive outcomes, AI technologies and their subdomains are being implemented in healthcare delivery. The two main techniques for implementing AI are machine learning (ML) and deep learning (DL), which are terms that are frequently used interchangeably. Deep learning is a branch of machine learning. ML generates predictions by spotting patterns in data by means of mathematical algorithms. DL produces forecasts using multiple layers of fabric neural network algorithms that are modeled after the brain’s neural network architecture. In the past ten years, with advancements in big data, algorithms, computing power, and Internet technology, AI has excelled in numerous tasks across a wide range of industries, including identification of faces, image classification, speech recognition, automatic translation, and healthcare [4]. The main ML techniques are support vector machines (SVMs), decision trees, and K unsupervised algorithms, while the most commonly used for DL today are convolutional neural networks (CNNs) [5]. Figure 1 presents a few of the most basic ML and DL approaches.

ML fundamentally seeks to replicate or mimic humans’ capacity for pattern recognition. Traditional ML approaches take far longer to teach and test based on a specific problem than DL approaches. SVMs, decision trees, random forests, gradient boosting (such as XGBoost), and other conventional ML techniques are examples of traditional ML techniques. There is a significant flaw with decision trees, namely a decision tree divides samples extremely precisely, but dividing samples too precisely causes overfitting of the training set, and dividing samples coarsely results in a decision tree that does not fit the samples properly. Decision trees called random forests are based on the concept of learning and bagging combined. Two factors—the random selection of the dataset and the random selection of the characteristics utilized in each tree—reflect the unpredictability of a random forest the most. The XGBoost technique repeatedly constructs an ensemble of decision trees. The capacity of this technique to manage missing data, capture nonlinear correlations between the model features and the outcome, and have higher-order interactions between variables is its key benefits over conventional logistic regression-based risk models [7].

## Training artificial intelligence models

Several processes are necessary for training an AI model, including data gathering and preparation, model selection, model training, and hyperparameter tuning.

### Data collection and preprocessing

With the rapid development of modern medicine, various types of data are emerging. A large amount of imaging data has been generated as represented by X-ray, CT, and MRI, and the development of pathology has made sectioning the gold standard for tumor diagnosis. In addition to the traditional clinical information data, with the remarkable advances in sequencing technology over the past two decades, how to deal with the large amount of molecular data brought about by genomics, transcriptomics, proteomics, etc., has also become a matter of close attention for clinicians. Later, we will describe how to deal with a single type of data. However, a patient usually does not have only one type of test and one type of data, so we will also introduce how to integrate different types of data to enhance computational models.

To facilitate the subsequent model training, we need to preprocess these data. For digital data, we need to remove outliers, deal with missing values, and normalize the data. The most often utilized AI algorithms, using EHR as an example, are deep learning, decision tree algorithms, and regression algorithms. While completing regression tasks to finish disease risk prediction, researchers also use classification tasks to extract lesion characteristics from illnesses and categorize them [8]. The initial set of preparation measures mentioned above led us to normalize the data. To extract the characteristics from the data, we must then process it further. Digital data may be used directly as raw data.

To enhance the diversity of the dataset, we may need to adopt techniques such as pitch shifting [9], time stretching [10], and adding background noise for sound data. Sound data can have features extracted using methods such as Mel Frequency Cepstrum Coefficients (MFCCs). Deep learning approaches are already being used in numerous creative photoacoustic tomography projects for a range of goals, such as enhanced quantification, inadequate sampling modification, resolution enhancement, and reconstruction artifact removal [11]. The sort of cancer for which sound data are most frequently employed is skin cancer. In a previous study [12], vibrational optical tomography (VOCT) and machine learning were utilized to assess the specificity and sensitivity of employing light and audible sound to distinguish between skin malignancies and normal skin. An OQ LabScope 2.0 was used to measure the resonance frequency. Various machine learning techniques, including logistic regression, support vector, and decision-making models, were then used and contrasted to determine which model produced the best reliability. A recent study [13] imaged breast cells, especially malignant MDA-MB-231 cells and normal MCF10a cells, using phonon microscopy. A shallow convolutional neural network was trained to differentiate signals coming from healthy cells, malignant cells, and background using the raw phonon data as inputs. They used the Gramian angular summation fields approach to transform the signals into a format that was appropriate for the network, which produced visual representations of the time-resolved signals. The final model has a 93% accuracy rate.

For image data, the preprocessing process may involve techniques such as rotating, inverting, scaling, and adding noise. The best AI tool for processing images is deep learning. The most representative of these is convolutional neural networks (CNNs) [14–16]. A CNN often includes the following layers: an input layer, a convolutional layer, an activation layer, a pooling layer, and a fully connected layer. The core of CNN’s efficient image processing lies in the convolutional layer (Fig. 1.C). In this way, an image is digitized. Transformer neural networks have recently replaced convolutional neural networks (CNNs) in many nonclinical and clinical image processing jobs because of their enhanced reliability and efficiency in computer vision tasks [17, 18]. According to a previous study [19], transformer-based methods outperformed attention-based MIL techniques in terms of data efficiency since they were better at learning from tiny quantities of data.

Different data can also be converted to each other. In addition to converting image data to numerical values, image data can also be converted to sound data to acoustically differentiate between malignant and benign lesions [20]. In the last layer of the DL classifier, all 1024 nodes’ weighted activations were sonified—that is, data were represented using nonspeech to produce sounds—after training, fine-tuning, and data replenishment [21].

Indeed, multimodality is inherent in health data. Our current state of health comprises a multitude of data, ranging from the broad macro-level (disease existence or lack) to the detailed micro level (biomarkers, proteomics, and genomes). To improve prediction performance, a subsection of machine learning called “multimodal machine learning” seeks to create and train models that can use a variety of data sources and understand how to link to or integrate distinct modalities [22]. The majority of multimodal clinical decision-support systems in use today rely on an uncoordinated method of combining data from several sources [23–25]. IRENE was the first medical diagnostic transformer-based model to perform holistic representation learning on multimodal clinical data concurrently using a single, cohesive AI model [26]. In contrast to earlier nonunified approaches, IRENE avoids taking separate pathways for learning modality-specific characteristics in nonunified techniques, instead gradually learning holistic representations of multimodal clinical data. Large language models, which have just been developed, may improve this method [27].

### Model selection

Depending on the kind of data and the issue we are trying to address, we must select the best ML or DL architecture. When the dataset holds numeric data, we can use traditional regression models (e.g., linear regression) for prediction and traditional clustering algorithms (e.g., support vector machines (SVMs)) for classification. When the data we need to deal with are sound and image data, we need to choose to use neural networks (NNs), such as CNNs and RNNs, to help us mine the deeper features of images. If we also need to focus on the sequence information between the data, we can use long short-term memory (LSTM).

### Model training

Conventional model training is divided into two steps: training and verification. We can divide the existing dataset into a training dataset and a verifying dataset at a ratio of 7:3 or 8:2. We first use the training dataset to train the model so that the model automatically optimizes the parameters. To achieve better recognition and prediction results, then we use the verifying dataset to verify the training effect of our model.

### Hyperparameter tuning

In an AI model, parameters are often divided into two categories: hyperparameters and model parameters. Model parameters are parameters that can be automatically optimized through continuous training and iteration, while hyperparameters are fixed parameters that need to be set manually. The number of layers in the convolution layer of the CNN is one kind of hyperparameter. The setting of hyperparameters will directly affect the performance of a model. When the classification and prediction of a model is not good, we can modify the hyperparameters to provide its performance. The optimization of hyperparameters is complicated work that requires sufficient professional knowledge and experience accumulated from long-term tuning (hyperparameter tuning).

With the development of AI, an increasing number of models have been built, and Table 1 describes the latest FDA-approved AI models related to cancer.

**Table 1:** A list of oncology-related AI technologies with FDA approval

Device name	Year	Company	Task	Type	Clinical applications	FDA summary
Quantib Prostate	2022	Quantib BV	Offers features for reading prostate MRI in one workflow	Supervised machine learning algorithms	Retrospective analysis of 108 patients’ prostate mpMRI tests [28] was done	K221106.pdf (fda.gov)
EFAI RTSuite CT HN-Segmentation System	2022	Ever Fortune.AI Co., Ltd	Uses noncontrast CT scans, and reveals the first outlines of the organs that are at risk in the head and neck area	DL	The intended users are radiation oncology professionals, including but not limited to, radiation oncologists, medical physicists, and dosimetrists	K220264.pdf (fda.gov)
Precise Image	2022	Philips Medical Systems Nederland, B.V	Uses computer reconstruction to create images of the head and body using X-ray transmission data collected at various angles and planes	DL	Head, body, vascular, and cardiac	K210760.pdf (fda.gov)
Paige Prostate	2021	Paige.AI	Assists the pathologist in the diagnosis of prostate cancer	DL	A cohort of 105 prostate core needle biopsies (CNBs) was evaluated through digital pathology [29]	DEN200080.pdf (fda.gov)
Oncospace	2021	Oncospace, Inc.	Supports radiation oncologists and medical dosimetrists during radiotherapy treatment planning for prostate, thoracic, pancreas, and head & neck cases	ML	Oncospace has provided clinical performance testing results with a library comprising a clinical dataset to demonstrate safety or effectiveness	K202284.pdf (fda.gov)
VBrain	2021	Vysioneer Inc.	Assists in brain tumor contouring	DL	100 randomly selected patients with unresected brain metastases treated with SRS at an institution [30] from 2017 to 2020	K213628.pdf (fda.gov)
GI Genius	2021	Cosmo Artificial Intelligence—AI Ltd	Aids endoscopists in detecting colonic mucosal lesions	DL	COLO-DETECT was the first multicenter randomized controlled trial evaluating GI Genius™ in real-world colonoscopy practice [31]	K211951.pdf (fda.gov)
EndoScreener	2021	Chengdu Wision Medical Device Co., LTD	Automatically detects polyps	DL	A prospective, multicenter, single-blind randomized tandem colonoscopy study of 196 patients[32]	K211326.pdf (fda.gov)
NinesMeasure	2021	Nines, Inc.	Measures the size of selected pulmonary nodules in a radiological image	ML	The device is intended to be used as a measurement tool by a trained radiologist and is limited to analysis of imaging data	K202990.pdf (fda.gov)
MammoScreen	2020	Therapixel	Identifies breast soft tissue lesions or calcifications	ML	A cross-sectional study of 448 invited participants demonstrated its effectiveness [33]	K211541.pdf (fda.gov)
PROView	2020	GE Medical Systems SCS	Provides an automatic segmentation of the prostate on MRI T2-weighted acquisitions	DL	It is a tool that aids clinicians in reviewing multiparametric prostate magnetic resonance (MR) images following PI-RADS guidelines	K193306.pdf (fda.gov)
AI-Rad Companion (Pulmonary)	2019	Siemens Medical Solutions USA, Inc.	Supports quantitative and qualitative analysis of previously acquired CT DICOM images	DL	499 radiographs were retrospectively included to evaluate the performance of the AI-Rad [34]	K183271.pdf (fda.gov)
ProFound AI Software V2.1	2019	ICAD Inc.	Detects malignant soft tissue densities and calcifications in digital breast tomosynthesis (DBT) images	DL	The detections and “certainty of finding” and case scores assist interpreting physicians in identifying soft tissue densities and calcifications	K191994.pdf (fda.gov)
QuantX	2017	Quantitative Insights, Inc.	Registers images and has automated segmentation and analysis functions	ML	Provides features to aid radiologists in the visualization and analysis of breast MR images	K170195.pdf (fda.gov)

We describe the types of FDA-approved AI models, clinical applications, tasks, and provide links

ChatGPT, a public and open research preview that was released in November 2022, quickly popularized OpenAI’s work with autoregressive LLMs based on generative pretrained transformers (GPT). Tiffany H. Kung et al*.*[35] evaluated ChatGPT on the United States Medical Licensing Examination (USMLE) and found that ChatGPT performed at or near the passing threshold of 60% accuracy. According to their study, LLMs such as ChatGPT may be able to help human students in a medical education context as a step toward eventual inclusion in clinical decision-making.

However, before clinical decisions are made, physicians often perform an additional and crucial step wherein they ask patients a series of questions to further clarify issues and schedule relevant tests to obtain more accurate information to support a diagnosis. This step is currently difficult for ChatGPT to accomplish proactively. We must recognize that AI’s purpose is not to eclipse or take the place of humans but rather to offer decision-support tools that aid in the clinical management of cancer patients by medical professionals and researchers studying the illness.

## Increasingly significant role of AI in tumor diagnosis, staging, and grading

### Tumor screening and early detection

An important way to reduce cancer incidence and mortality is through screening in a population. With the increasing awareness of health screening, an increasing number of smart detectors are being invented to improve the early detection of cancer. For the purpose of early cancer diagnosis, traditional machine learning (ML) approaches including random forest (RF), naïve Bayes, k-nearest neighbor, support vector machines (SVM), and related methods have been applied. Convolutional neural networks are the most commonly used model in image-based screening, and SVM algorithm-based and mass spectrum-based feature selection are commonly used in molecular diagnostics.

Digital breast tomosynthesis (DBT) can improve breast cancer detection rates by decreasing recall rates, increasing incremental cancer detection rates, and increasing cancer detection rates [36–38]. However, DBT images take longer to interpret [39]. An AI model [40] was built consisting of a collection of 50 different classifiers. The clinical data and data from the Digital Imaging and Communications in Medicine tags were analyzed by five machine learning (ML) classifiers, and the four DBT viewpoints were processed by 45 deep learning (DL) classifiers. The ability of the AI model to recognize common digital breast tomosynthesis screening techniques reduced the number of examinations that required doctors interpretation in a simulated clinical workflow.

For lung cancer screening, X-rays and low-dose CT are the most routine screening methods. DL algorithms have made good progress [41–43] in improving X-ray screening of lung nodules. However, low-dose CT is more accurate than X-rays. The use of low-dose spiral computed tomography (CT) scans has been shown to significantly reduce lung cancer mortality [44]. A CNN (CXR-LC) was created utilizing information that is frequently found in electronic records (CXR picture, age, sex, and whether or not a person is a smoker) and validated that it can identify smokers at high risk of developing incident lung cancer in two large lung cancer screening trials (PLCO, NLST) [45, 46]. A DL system [47] was created that can correctly identify the existence of lung cancer within three years and account for all pertinent nodule and nonnodule markers on screening chest CTs. Their research was the first to create a deep machine learning prediction method without the use of computer-aided diagnostic tools to assess a person’s 3-year probability of developing lung cancer and related lung cancer-specific mortality. Kiran Vaidhya Venkadesh et al*.* [48] created and externally verified a CNN-based DL algorithm for estimating the likelihood of malignancy in lung nodules found by low-dose screening CT, which demonstrated good performance, on par with thoracic radiologists, at estimating the malignancy risk of pulmonary nodules observed during screening CT(AUC = 0.93). However, their researches included a number of restrictions. Firstly, one CT scan was employed in the created method, and a prior CT image was not taken into account [48]. Secondly, on average, members of the cohort [47] had LDCTs for screening every year, which may cause bias in the measurement results.

In order to address the above issues, a deep learning system was developed that can forecast the probability of developing lung cancer six years from now. Newly developed Sybil [49] can precisely estimate a person’s future risk of lung cancer on a single LDCT scan, enabling more individualized screening. When using CNNs to perform lung nodule classification, data imbalance is a crucial issue to be considered. To address this, MLSL-Net [50] was established, which employs multilabel softmax loss (MLSL) as the performance index. Recently, Xiangde Luo et al*.* [51] proposed a centroid matching detection network (SCPM-Net) based on a 3D sphere representation to address the limitations of CNNs, namely that they have limited elasticity when dealing with pulmonary nodules that have a large range of sizes and require predefined anchor parameters, such as the size, number, and aspect ratio of anchors. According to experimental findings on the LUNA16 dataset, the SCPM-Net framework has an average sensitivity of 89.2% at 7 preset FPs/scan.

In addition to imaging, molecular tests are an important part of early screening. Nine lipids have been identified [52] as the features most crucial for early-stage cancer detection using SVM algorithm-based and mass spectrum-based feature selection. The chosen lipids were found to be differentially expressed in in situ early-stage lung cancer tissues according to matrix-assisted laser desorption/ionization MS imaging. A diagnostic screening approach for gliomas called DeepGlioma [53] uses deep neural networks and stimulated Raman histology (SRH) to quickly screen for molecular changes in newly collected glioma specimens.

Adenomas of the colorectum have been shown [54] to be highly correlated with colorectal cancer. Several studies have recently developed different AI models for improving adenoma detection rates [32, 55–62]. To predict the polyp class, two DL models, SEG and noSEG, were trained using 3D CT colonography image subvolumes. Model SEG was also trained using polyp segmentation masks [56]. Joel Troya et al*.* [58] combined side optics with AI. Hong Xu et al*.*[60] invented an AI polyp detection system (Eagle-Eye) with real-time notification on the same monitor of the endoscopy system. All of these models have been shown to enable CT colonography to noninvasively distinguish benign and premalignant colon polyps. In addition, AI has been shown to save on the cost of colonoscopies [62, 63]. Along with colonoscopy, noninvasive diagnostics, including plasma fluorescence [64], tests for intestinal microbiota [65], and spatial light interference microscopy [59], can be used in conjunction with AI to enhance the early detection of colorectal cancer.

**Fig. 2:**
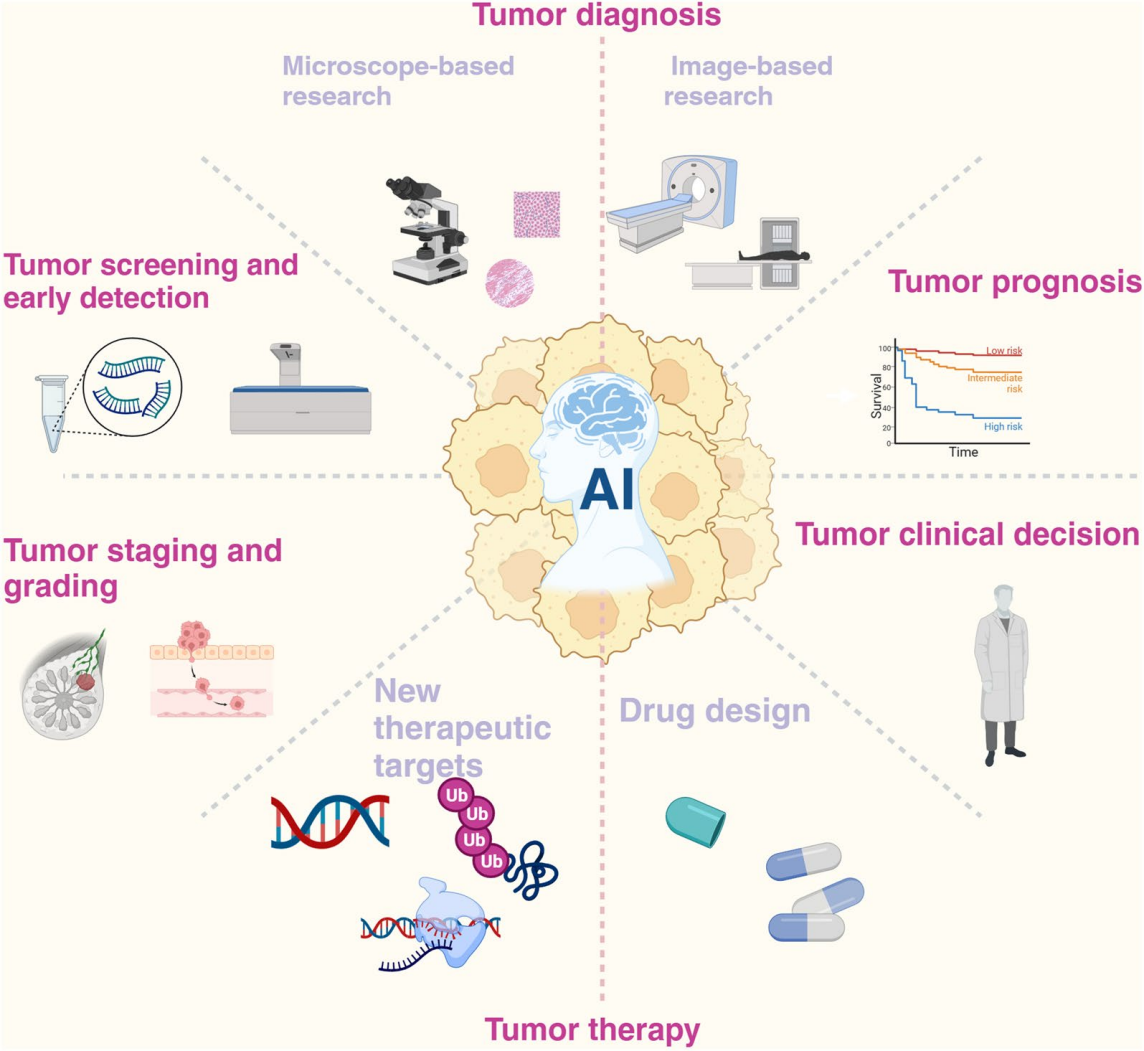
AI in oncology, including early screening, diagnosis, treatment, prognosis, and clinical decision-making. (Created with BioRender.com)

Furthermore, cervical cancer [66], skin cancer [67, 68], oral cancer [69, 70], esophageal squamous cell carcinoma and adenocarcinoma of the esophagogastric junction [71] can also be detected and distinguished early using AI models. The above studies greatly demonstrate the potential of AI models in detecting early cancers. Figure 2 describes the function of AI in cancer.

## Tumor diagnosis

When assessing a patient’s signs and symptoms, clinicians typically draw on their own knowledge and professional expertise. Given the enormous amount of clinical data, it can be challenging for them to make a diagnosis quickly. In addition, there are issues with individualized patients, atypical test results, and false negatives. Doctors with heavy clinical workloads frequently run the risk of missing or misdiagnosing patients. However, AI can process a large amount of data in a short period and can improve the accuracy and speed of disease diagnosis, thus allowing AI to be widely used in cancer diagnosis.

The current approaches of AI for cancer diagnosis can be routinely divided into two main types: microscopy-based and image-based AI. Microscopy-based AI mainly explores models to improve the correct diagnosis from a histopathological point of view. Image-based AI involves algorithms that reduce the incorrect diagnosis rate from images such as X-ray and CT scans.

Here, we focus on the latest advances in current conventional microscopy-based and image-based AI for cancer diagnosis. Weakly supervised learning models and generative adversarial networks are the most relevant models for histopathology. Various models in deep learning play an important role in assisting imaging to diagnose tumors.

### Microscopy-based research

Pathological diagnosis was once considered the gold standard for cancer diagnosis, but errors inevitably exist [72]. In the past, the majority of techniques relied on morphological traits or hand-crafted features to identify malignant and noncancerous cells in histopathological images [73, 74]. The power of AI is not limited to image categorization, where the goal is to forecast a certain condition consistent with the image. Generic models replicate the original visuals and provide fresh possibilities, such as quick and safe model training. Curating enormous databases of digitized tissue sections has been made practical by whole-slide imaging (WSI) of tissues, affordable storage, and rapid network data transfer [75]. Annotation-less techniques have gained popularity in recent high-profile papers [27, 76, 77]. These approaches do not rely on annotations for individual structures such as nuclei, cells, or tissues; instead, they simply need one label per complete WSI, such as malignant/benign, which characterizes the WSI as a whole. AI techniques leverage weak labels by utilizing the multiple instance learning (MIL) AI framework [78].

Histological staining, a vital step in the pathology workflow, is required to offer tissue contrast and color by permitting chromatic discrimination between different tissue components. The most popular stain, hematoxylin and eosin (H&E), sometimes known as the “standard stain,” is used in almost all clinical settings [79]. Aman Rana et al. [80] trained conditional generative adversarial networks (cGANs), which automatically convert native nontrained RGB WSIs to computational H&E-stained pictures. Binglu Huang et al*.* [81] collected 1037 H&E-stained pathology images from 2333 GC patients to develop GastroMIL, which achieved an accuracy of 0.920 in an external validation set, superior to that of junior pathologists and comparable to that of expert pathologists. It is challenging to identify mitosis in H&E-stained slices because there are few datasets available and because mitotic and nonmitotic cells are similar. Comparing performance metrics of multi-CNN combinations with other classifiers such as AdaBoost and random forest, multi-CNN combinations with three pretrained CNNs and a linear SVM have been shown to provide 93.81% accuracy and a 92.41% F1 score for detecting mitosis [82].

DL can also predict biomarkers with high performance from cancer pathology slides. Malignant cancer cells are created when normal cells have oncogenic driver mutations, which completely alter the behavior of the cells by rewiring their internal systems [83]. When genetic mutations are present, the genotype as established by an enzyme-mediated biological research assay or other gold standard testing is used as the ground-truth approach during the traditional diagnostic workup to identify the picture label. The term “ground truth” describes the kind of test that is employed to identify training pictures. As a result, by examining histological image data, the DL classifier may be trained to replicate the “ground truth.” In contrast to basic DL applications, these progressed applications for deep learning can give doctors extra information that is not being gleaned from routine material in the current medical workflows. They signify a novel category of biomarkers possessing prognostic and/or predictive utility. Microsatellite instability (MSI) due to mismatch repair (MMR) defects accounts for 15–20% of colon cancer (CC), and many DL algorithms [84–86] have been established to detect MSI. To predict additional biomarkers for CRC from pathology slides, Jan Moritz Niehues et al*.*[14] comprehensively assessed six distinct cutting-edge DL architectures. They discovered that while MSI and BRAF mutant prediction was performed at a clinical-grade level, PIK3CA, KRAS, and NRAS mutation prediction did not meet these standards. An algorithm for cell-distance analysis of multiplex fluorescence immunohistochemistry (mfIHC) staining and a framework for automated Ki-67 LI quantification were created and validated in a cohort of 12,475 prostate cancer samples in order to enable automated Ki-67 LI assessment in common clinical practice [87]. AI-assisted analysis of biomarkers in thyroid cancer [88], and breast cancer [89] also helps in accurate diagnosis.

Overall, these models may be useful for diagnosing and categorizing malignancies if their performance is supported by prospective studies. This is especially true given that their performance is on par with or even superior to that of experts in the area.

### Image-based research

AI has much potential for helping radiologists with their work and for image information mining. Clusters of graphics processing units are integrated into high-performance computers, which have powerful computational power. In addition to the AI we mentioned above, which can assist in early screening for breast, lung and colorectal cancers, some other promising imaging tests can be combined with AI to improve diagnostic accuracy.

Numerous modalities have been used to acquire vast numbers of high-quality skin photos, exploiting the exceptional advancements in optical imaging methods. Therefore, AI has made promising progress in the detection of skin cancer through dermoscopy. Inception V3 models that have already been trained have been used [90] to classify skin lesions and to present dermatologist-level prediction outcomes. The knowledge distillation approach is also often used to help diagnose melanoma [91, 92]. In addition to the simple teacher–student model, the SSD-KD approach [93], a unique self-supervised diversified knowledge distillation technique, has been used for the lightweight multiclass categorization of skin diseases utilizing dermoscopy images. In that study, the conventional single relational modeling block was substituted with dual relational blocks in terms of technological innovation. Multi-Site Cross-Organ Calibrated Deep Learning (MuSClD), a novel approach to cross-organ calibration between two sites of digitalized histopathology images, was validated in nonmelanoma skin cancer. 3D images [94, 95], EfficientNet [96, 97], genetic programming (GP) [98], and new AI algorithms on smartphones [99, 100] have also been developed for skin cancer diagnosis.

To supplement human visual inspection, AI can assist in the detection of undetectable tumor lesions on PET scans. Ga-PSMA-11 PET-based radiomics features have been used to generate random forest models that accurately predicted invisible intraprostatic lesions [101]. Biopsy and magnetic resonance imaging (MRI) are frequently used to diagnose intracranial tumors. Due to the similar phenotypes of various tumor classes on MRI scans, it has been difficult to identify tumor types, especially rare types, from MRI data. A DL method for segmenting and classifying 18 distinct types of intracranial tumors was developed [102] using T1- and T2-weighted images and T2 contrast MRI sequences and evaluated with an AUC of 0.92.

AI may easily be applied to medical imaging, and major advancements in this area have been made in recent years. AI eliminates the uncertainty that people contribute to decisions and delivers objective measurements for each choice. However, the limits are also readily apparent. The molecular causes of illnesses are not revealed by morphological evidence. By using this method, disease states with the same morphological appearance cannot be discriminated.

## Tumor staging and grading

Important factors for tumor T-staging include the size and degree of invasiveness of primary tumors, which comprise descriptions of their shapes. Convolutional neural networks are most used in this task. The T stage of Barrett’s carcinoma is a crucial consideration when choosing a course of therapy. Endoscopic ultrasonography is still the norm for preoperative staging, but its usefulness is under question. To help with staging and to improve outcomes, new tools are needed. With a high accuracy of 73% in diagnosing esophageal cancer, an AI system built around endoscopic images has been developed [103]. Tumor sizes and forms vary, making individual slice-by-slice screening for T-staging time intensive. Consequently, a multi-perspective aggregation network (TSD Net) has been created with ideas from oncological diagnostics that included different diagnosis-oriented knowledge and enabled automatic nasopharyngeal carcinoma T-staging identification [104].

Advances in imaging histology have greatly contributed to helping TNM staging of tumors. Separate iterations of the machine learning models have been created using both the entire collection of extracted features (full model) and just a selection of the previously discovered robust metrics (robust models) to confirm that CT-based radiomics signatures were effective tools for determining the grade and stage of ccRCC [105]. Additionally important in the early phases of decision-making, but time-consuming, is a delineation of the tumor. To forecast the grade of a tumor while also segmenting it, a single multi-task convolutional neural network has been created using the whole 3D, structural, preoperative MRI data [106].

Accurate assessment of lymph node metastasis (LNM) is essential for evaluating the staging and grading of tumor patients. In addition to offering a straightforward “yes” or “no” response on the likelihood of having cancer, AI models can also identify the disease site from a test picture. One of the most common applications is to help find the localization of metastatic tumors. Using whole-body PET/CT scans, convolutional neural networks (CNNs) based on UNet [1] were trained to detect and separate metastatic prostate cancer lesions fully automatically [107]. The localization of tumor metastasis in whole-slide images has also been studied extensively in recent years [107–110]. The condition of the lymph nodes (LNs) prior to surgery is crucial for the management of colorectal cancer (CRC). With areas under the curve (AUCs) of 0.79, 0.73, and 0.70 in the training set, testing set, and verification set, respectively, a deep learning (DL) model [111] with features gathered from improved venous-phase CT images of CRC has been proposed to identify LNM in CRC. Shaoxu Wu et al*.* [112] created a diagnostic algorithm called LNMDM based on AI that was effective for finding micrometastases in lymph nodes and was demonstrated not only in bladder cancer (0·983 [95% CI 0·941–0·998]) but also in breast cancer (0·943 [95% CI 0·918–0·969]) and prostate cancer (0·922 [95% CI 0·884–0·960]). AI plays a significant role in aiding diagnostics to find lymph node metastases in slide pictures. Lymph node metastases, especially micrometastases, were successfully identified by the LNMDM [112] on whole-slide images in bladder cancer. The VIS AI algorithm demonstrated comparable accuracy and NPV in identifying LN metastases on breast cancer. In summary, the implementation of AI in tumor staging and grading has significantly improved tumor prognoses and increased the general survival rate of cancer patients.

## Tumor therapy

### AI for exploring tumor therapeutic targets

**Fig. 3:**
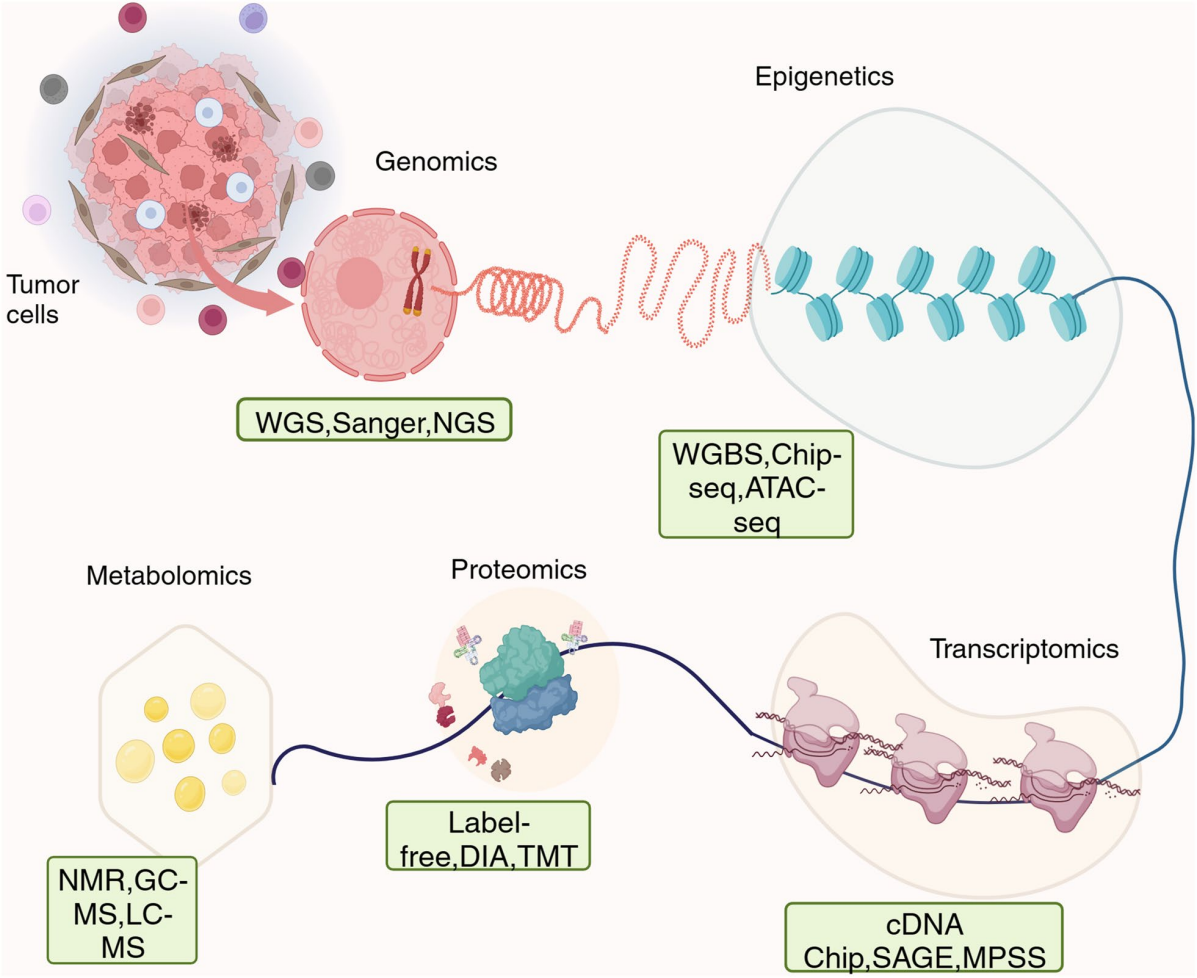
Components of multiomics and the main techniques. The combination of AI and *multiomics* has led to the discovery of new targets for cancer therapy. (Created with BioRender.com)

In recent years, the development of multiomics technologies in cancer research [113, 114] has greatly facilitated the discovery of anticancer targets [115–117]. The advancement of precision medicine and translational medicine will be significantly aided by the use of ML and DL to mine multiomics data to investigate complicated disease causation processes and treatment response mechanisms. In the following, we describe in detail the advances in genomics, epigenetics, transcriptomics, proteomics, metabolomics, and multiomics in cancer target discovery. Figure 3 describes the main sources of these six components and the advanced methods currently comprising them.

### Genomics

The genome contains inherited information that controls gene expression to shape the structure and working machinery of the cell [118]. Genomics focuses on understanding the composition, organization, visualization, and modification of an organism’s whole genome [119]. The rise of the genomic era has also boosted precision medicine and cancer [120]. The approach of a meta-learning model [121] allows users to discover significant pathways in cancer and priority genes based on their contribution to survival prediction. To fully understand how cancer develops, progresses, and is treated, accurate somatic mutation detection is difficult yet essential. The first method for detecting somatic mutations based on deep CNNs is called NeuSomatic [122]. However, the fact that matched normal specimens are not frequently acquired in clinical practice is a major barrier to genetic testing in cancer. The somatic vs. germline status of each discovered change may be predicted using SGZ, [123] which does not need a patient-related standard control, by modeling the mutation’s allele frequency (AF), accounting for the cancer content, cancer ploidy, and local copy number. Similarly, a recently created method, Continuous Representation of Codon Switches [124] (CRCS), a DL-based technique, can aid in the identification and investigation of driver genes as well as the detection of cancer-related somatic mutations in the absence of matched normal samples.

Taking colon cancer as an example, numerous studies [125–128] have subtyped colorectal cancer based on similar and different biological traits and pathways, and they have identified the relationships between these pathways and patient prognosis, overall survival, and responsiveness to various treatments—particularly targeted therapy and immunotherapy. Using 499 primary colorectal neoplasm diagnostic images from 502 individuals in The Cancer Genome Atlas Colon and Rectal Cancer (TCGA-CRC-DX) cohort, a retrospective study established a weakly supervised DL framework incorporating three separate CNN models [85]. After comprehensive validation, the method was shown to be helpful for patient classification for targeted medicines, with possible cost savings and quicker turnaround times compared to sequencing- or immunohistochemistry-based techniques. The research, however, examined each individual image tile without considering the significance of the spatial relationship between tiles. In a recent study [129], a method for forecasting cross-level molecular profiles involving gene mutations, copy number variations, and functional protein expression from whole-slide pictures was proposed. This method focuses on the spatialization of cancer tiles. In the training dataset, the model performed exceptionally well in predicting a variety of genetic alterations and then identifying targeted therapies for colon cancer patients.

### Epigenetics

Epigenetic modification is the genetic change in the way genes operate and express without altering the DNA sequence. DNA methylation, histone modification, and chromatin structure manipulation are the three primary epigenetic modifications that are now understood [130]. Although there are high-quality data on DNA methylation, few samples have RNA-seq data due to numerous experimental difficulties. Therefore, an innovative technique called TDimpute [131] was created to reconstruct lost data on gene expression from DNA methylation data using a transfer learning-based neural network. Understanding how epigenetics regulates gene expression to govern cell functional heterogeneity is dependent on the ability to predict differentially expressed genes (DEGs) from epigenetic signal information. On the basis of epigenetic data, a multiple self-attention model (Epi-MSA) [132] was suggested to predict DEGs. To determine which gene locations are crucial for forecasting DEGs, Epi-MSA first applies CNNs for neighborhood bin information embedding and then makes use of several self-attention encoders on various input epigenetic parameters.

### Transcriptomics

Transcriptomics is a useful tool for comprehending the physiology of cancer and locating biomarkers. It includes analyses of alternative transcription and alternative polyadenylation, detection of integration transcripts, investigations of noncoding RNAs, transcript annotation, and finding novel transcripts [133]. One study using DL algorithms to interpret common cancer transcriptome markers [134] showed that across a wide range of solid tumor types, dysregulation of RNA-processing genes and aberrant splicing are widespread traits on which fundamental cancer pathways may converge. Molecular pathology plays an important role in cancer, but whether it is possible to estimate the levels of gene expression based on a visual inspection of H&E-stained WSIs has never been thoroughly explored. Numerous studies have been conducted to predict cancer gene expression, including that of prostate [135] and breast [136] cancers, across the transcriptome from histopathological images. A DL model called HE2RNA [137] based on a multitasking poorly supervised technique was created using matched WSIs and RNA-Seq profiles from TCGA data, which included 8725 patients and 28 distinct cancer types. This increases the likelihood of discovering novel gene targets. Patients’ responses to treatment are significantly influenced by the quantity, composition, and geographic distribution of the cell groups in the tumor microenvironment (TME) [138]. The thorough characterization of gene regulation in the TME has been made possible by recent developments in spatial transcriptomics (ST) [139, 140]. Three new approaches have recently been developed: Kassandra [141], XFuse [142], and TESLA [143]. Kassandra is a tree ML algorithm that was taught to precisely rebuild the tumor microenvironment (TME) using a large database of > 9,400 tissue- and blood-sorted cell RNA profiles combined into millions of artificial transcriptomes. According to Kassandra’s deconvolution of TME components, these populations play a part in tumor etiology and other biological processes. By utilizing data from H&E-stained histological images, XFuse predicts superresolution gene expression per pixel. TESLA is an ML framework that incorporates gene expression and histological image data into ST to study the TME. The innovative aspect of TESLA is the annotation of diverse immune and tumor cells on histological images directly.

In addition, the identification of lncRNAs [144–146] and microRNAs [147, 148] by ML can assist in the precise treatment of cancer. In the fight against cancer, therapeutic decisions are increasingly based on molecular tumor features, and cancer tissue molecular profiling is becoming an essential component of standard diagnosis [149]. To reduce individualized patient differences, scGeneRAI [150] uses layerwise relevance propagation (LRP), an explainable AI technique, to extrapolate individual cell gene regulation networks from single-cell RNA sequencing data. Oncology drug response is a major challenge in cancer treatment. With an average Matthew correlation coefficient (MCC) and AUC of 0.56 and 0.80, respectively, the classification and regression tree (CART) model from interpretable ML models has proven to be the best model for predicting how breast cancer would react to doxorubicin [151]. At the single-cell level, ScDEAL is a deep transfer learning system that integrates bulk cell-line data to predict cancer medication response at the single-cell level. Finding drug resistance targets at the level of transcriptional profiles using AI deserves more research in the future.

### Proteomics

Proteomics is a broad study of proteins that identifies and counts the proteins present in a biological sample, such as a sample of cells, tissues, or bodily fluids. Proteomics data offer the benefit of providing a numerical number of individual proteins throughout the body and dynamic characteristics that develop over time and among individual subjects, in contrast to other forms of omics data, such as genomic data. Mass spectrometry (MS) is a key tool used in proteomics research [125]. MS-based proteomics has advanced quickly in terms of lower cost and higher throughput, regularly permitting large-cohort studies with tens of thousands of participants and tens of millions of identified proteins in cancer cells and other biological samples. However, the majority of research concentrates on the final proteins discovered using a collection of algorithms that compare partial MS spectra with the ordered database, leaving the problem of pattern identification and categorization of the raw mass-spectrometric information unanswered. Consequently, for the analysis of massive MS data using deep neural networks (DNNs), the publicly available MSpectraAI [152] platform and the tumor classifier [153] have been developed, which could expand the intriguing use of DL techniques for classifying and predicting proteomics data from multiple cancer types and distinguishing between tumor and nontumor samples.

Sequential Window Acquisition of all Theoretical Mass Spectra-MS (SWATH-MS) is a cutting-edge MS method that enables the measurement of nearly all peptides as well as proteins present in a single sample, making it valuable in research involving massive sample cohorts [154]. It can be used to facilitate the categorization of CRC molecular subgroups and promote both diagnostics and the creation of novel medications [155]. Regarding colorectal cancer, a mechanism-based ML approach [156] has been proposed to find genes and proteins with substantial correlations to event-free patient survival and predictive potential to account for patient-specific variations in STN activity by building three linear regression models. The development of proteomics has contributed to the discovery of new targets in hematological tumors. Targetable enzyme characteristics have been revealed by proteomics of acute lymphoblastic leukemia that is resistant to Notch1 suppression [157]. Through the induction of long-lasting immune responses, T cells play critical roles in human defense against hematological tumors. In recent work [158], ML and nanoscale proteomics were coupled to subtype T cells in peripheral bloodstreams from single individuals with multiple myeloma. To reduce the possibility of overfitting the ML models, differentially expressed proteins (DEPs) were selected according to statistical significance, and only the top 13–15 DEPs were utilized. Thus, this work helped identify new targets for immunotherapy. Another DL network [159] identified the 20 proteins most strongly associated with FLT3-ITD in acute myeloid leukemia. In addition, DL and ML have been applied to proteomics data for pancreatic cancer [160] and diffuse large B-cell lymphoma [161] patients, respectively.

### Metabolomics

Metabolomics is a burgeoning area of research that utilizes technologically sophisticated analytical chemistry to perform high-throughput characterization of metabolites in cells, organs, tissues, or biological fluids [162]. New therapeutic targets have been suggested to target metabolic constraints in cancer as a result of metabolomics studies, which have revealed potential medicinal weak points for treating cancer [163]. Lipidomics is a branch of metabolomics that aims to study and analyze the lipids in the metabolome and the molecules that interact with them [164]. Metabolomics analysis can be performed using GC‒MS and LC‒MS, and LC‒MS is commonly used for the analysis of lipidomics. The combination of metabolomics and AI has flourished in various areas of cancer, including breast cancer [165, 166], head and neck cancer [167], colorectal cancer [168, 169], glioma cancer [170], esophageal cancer [171, 172], lung cancer [52, 173], kidney cancer [174], and neuroendocrine tumors [175]. With the greatest prediction accuracy (AUC = 0.93) and a deeper understanding of disease biology, a DL technique has been shown to be beneficial for metabolomics-based breast cancer ER status categorization [176]. By biologically interpreting the first hidden layer, this technique can identify eight frequently enriched crucial metabolomics pathways (adjusted P value 0.05) that cannot be identified by other ML techniques [176].

### Multiomics

Multiomics data, which include genomics, epigenomics, transcriptomics, and proteomics data, can offer profound information on the quantity and/or change in biological molecules across numerous dimensions in different tissues or cells [177]. Multiomics data have gained interest recently for their potential to offer a complete picture of patients, but their high dimensionality makes them difficult to use [178]. AI related to cancer multiomics has boomed in the last year and has strong potential for development in cancer therapy. Cancer driver genes are important targets in tumor therapy [179]. When compared to real tumors, an ML multiomics study [180] indicated carcinoma driver dysregulation in pancancer lineages of cells. Using graph convolutional networks to identify cancer driver genes is currently a popular research direction. DGMP [181] and MODIG [182] were created separately by applying pancancer multiomics data (including DNA methylation, copy number variation, mutation, and gene expression data). DGMP joins a directed graph convolutional network (DGCN) and multilayer perceptron (MLP), and MODIG is based on a graph attention network (GAT). They both have been shown to effectively identify cancer driver genes. Accurate tumor druggable gene discovery advances precision cancer therapy and deepens the comprehension of targeted cancer therapy. To determine the landscape of the genes that are capable of causing cancer, DF-CAGE [183], a novel ML-based method, combined the data from over 10,000 TCGA profiles on somatic mutations, copy number variations, DNA methylation, and RNA-Seq. DF-CAGE identified 465 putative cancer-druggable genes out of the approximately 20,000 protein-coding genes. These results provide insight into current pharmacological research and development efforts. DeepInsight-3D [184], which depends on the translation of structured data into images and then makes use of CNNs, represents a solution to the issue of the high dimensionality of the datasets combined with the lack of sufficiently large numbers of annotated samples in multiomics data. Future research toward better personalized treatment plans for various malignancies may be aided by the suggested enhancements.

The prognosis for non-small cell lung cancer (NSCLC), a heterogeneous illness, is dismal. A recent study [185] used ML models to develop a classification method and identified five novel NSCLC clusters with different genetic and clinical characteristics. Similarly, a multiomics data-affinitive AI algorithm [186] was created to identify new biomarkers in NSCLC but differently based on the graph convolutional network. Filippo Lococo et al*.* integrated multiomics and AI data into clinical trials, promoting better care for lung cancer patients [187]. The clinical significance of IMMT in KIRC has been validated using a combination of supervised learning and multiomics integration [188]. The majority of prognostic models for colon cancer are based on single-pathway genes. In a recent study [189], the molecular mechanisms causing the aggressiveness, recurrence, and advancement of colon cancer were explained using an integrative multiomics study, and ML methods were used to recognize the subtypes.

AI models can also help locate tumor sites during surgery. One of the most common applications is to help find tumor locations during surgery. The location, quantity, and size of cancer are critical factors for precise tumor excision, particularly in surgical patients. A study presented a novel double branch attention-driven multiscale learning method for MRI-based prostate and prostatic cancer segmentation networks [190]. The Dice similarity coefficients (DSCs) for prostate and prostate cancer MRI segmentation were 91.65% and 84.39%, respectively. Using magnetic resonance imaging, UNet +  + can automatically distinguish between liver tumors and normal hepatic tissue [191].

AI models can also help with tumor type classification. Neurosurgical cancer resection is the primary therapeutic method most frequently used for central nervous system (CNS) cancers. The kind of cancer is a crucial determinant in deciding whether the risk of a more vigorous excision is acceptable. A patient-independent transfer-learned neural network called Sturgeon was recently created to allow for the molecular subclassification of tumors of the central nervous system using sparse data [192]. In another study [193], after first-level categorization determined whether the aberrant area of the picture was a brain tumor, deep residual network (DRN)-enabled RDTDO was used for brain tumor classification, which was provided via second-level classification.

### Tumor prognosis

Clinical oncologists rely heavily on prognosis prediction to guide treatment choices by providing information on the predicted course of the disease and the chance of survival [194] (Table 2). The Cox proportional hazard regression model is used most frequently to predict survival [195]. However, due to its linear nature [196], the complex relationships between some features are difficult to interpret, which is compensated by the current survival models of ML and DL [197–199]. Common models for ML are SVMs, logistic regression, random forest, CatBoost, LightGBM, and XGBoost. SVMs are one of the most widely used algorithms in ML for cancer prognosis. In recent research [200], 265 surgical resection patients were included (training cohort: 212, internal validation cohort: 43). An SVM model was created using nine clinicopathological characteristics. Their SVM-based model may be utilized to forecast OS and DFS in GC patients as well as the advantages of adjuvant treatment in TNM stage II and III GC patients. Another study [201] fed each feature set selected by LASSO into three classifiers, namely SVM, hist gradient boosting (HGB), and XGBoost (XGB), to develop predictive models. In a study of breast cancer, SVMs and random forests were utilized as ML classifiers, while principal component analysis (PCA) and variational autoencoders (VAEs) were employed as reduced-dimensionality approaches [202]. However, multimodal classifiers were not proactively prospectively evaluated on original data in the study. RSF outperformed COX and SVM by a wide margin in research on GBM [203].

**Table 2:** Prognostic application of AI in different tumors

Type of cancer	Reference	Method	Year	Study population	Features and limitations	Performance
Breast cancer	Li et al. [208]	GBDT (XGBoost)	2023	SEER(2010–2019)	Focus on breast cancer brain metastases (BCBM)	3-year survival AUC = 0.803
	Li et al. [209]	SVM, CoxBoost	2023	TCGA	Establish robust and valid ROS signature (ROSig) to aid in assessing ROS levels	C-index: 0.736 for TCGA; 0.545 for Metabric
	Verghese et al. [210]	FCN	2023	Hospital	Capture systemic immune features in lymph nodes	Dice coefficient of 0.86 and 0.74 for capturing GCs and sinuses, respectively
	Li et al. [211]	RSF	2022	TCGA	Construct a novel hypoxia- and lactate metabolism-related gene signature	5-year AUC: 0.638
	Wang et al. [212]	CNN	2022	ClinSeq, TCGA, SöS-BC, SCAN-B	Improve breast cancer histological grading	Hazard ratio [HR] 2.94, 95% confidence interval [CI] 1.24–6.97, *p* = 0.015
Lung cancer	Ding et al. [213]	CNN(ResNet)	2023	Hospital	Assist pathologists in classifying histological patterns and prognosis stratification of LUAD patients	AUC: 0.93
	She et al. [214]	Feed-forward deep neural network	2020	SEER	Explore the lack of studies on the performance of a deep learning survival neural network in non-small cell lung cancer (NSCLC)	C statistic = 0.739 vs. 0.706
	Hosny et al. [215]	CNN	2018	Hospital	Deep learning networks may be used for mortality risk stratification based on standard-of-care CT images from NSCLC patients	AUC: 0.70 in radiotherapy; AUC: 0.71 in surgery
Colorectal cancer	Finn et al. [216]	Multinomial logistic regression, elastic net regression, and random forest	2023	SEER-Medicare registry	Extend the ability of claims-based research to risk-adjust and stratify by stage	95% CI, 0.43 to 0.46
	Kleppe et al. [217]	MIL	2022	Hospital	Integrate DoMore-v1-CRC and pathological staging markers to provide a clinical decision-support system	95% CI 6.39–17.93; *p* < 0.0001
	Bertsimas et al. [218]	RF, OPT	2022	Hospital	Provide a possible resolution to the long-standing debate on optimal margin width in CRLM	AUC: 0.76
	Kudo et al. [61]	ANN	2021	Hospital	Build a model to identify T1 colorectal tumors at risk for metastasis to lymph node and validate the model in a separate set of patients	AUC: 0.83
	Skrede et al. [219]	CNN	2020	Hospital	Develop a biomarker for patient outcome after primary colorectal cancer resection	95% CI 2·72–5·43; *p* < 0·0001)
Prostate cancer	Deng et al. [220]	CNN	2023	Hospital	Predict Ki-67 expression in prostate cancer	AUC: 0.939–0.993
	Saito et al. [221]	RSF, survival tree	2023	Hospital	Provide useful information for predicting the prognosis of metastatic prostate cancer	C-index: 0.64
	Lee et al. [222]	Cox proportional hazards, random survival forest, conditional inference survival forest, and DeepHit models	2021	SEER	Develop an improved prognostic model for predicting 10-year prostate cancer-specific mortality	C-index 0·829, 95% CI 0·820–0·838
Pancreatic cancer	Nimgaonkar et al. [223]	CNN (HoVer-Net)	2023	TCGA	This imaging analysis pipeline has promise in the development of actionable markers in other clinical settings where few biomarkers currently exist	95% CI [26.8, 63.9]
	Li et al. [224]	Random forest-based	2023	SEER	Explore two machine learning-based nomograms	3-year OS: AUC, 0.792 (95% CI: 0.717–0.949)
	Lee et al. [206]	Artificial neural network, logistic regression, random forest, gradient boosting, and support vector machine	2022	Hospital	Predict postoperative survival	2-year OS: AUC, 0.67; *p* = 0.35
Glioma	Voort et al. [106]	CNN	2023	Hospital	Noninvasively predicts multiple, clinically relevant features of glioma	AUC: 0.90
Skin cancer	Aung et al. [225]	ML	2022	TCGA and hospital	Evaluate the prognostic value of objective automated electronic TIL (eTIL) quantification	AUC: 0.793
Gastric cancer	Guan et al. [226]	SVM, RF	2023	Hospital	Evaluate and verify the predictive performance of computed tomography deep learning in gastric cancer	AUC: 0.9803
Oral cancer	Zhang et al. [227]	CNN	2023	Hospital	Identified OL patients with a high risk of OC development	HR = 4.52, 1.5–13.7
	Singh et al. [228]	SVM, naïve Bayes, decision trees, multi-Layer perceptron, logistic regression, and K means (unsupervised)	2022	PIK3CA, KRAS, TP53 and Gingival	Reveal key candidate attributes for GBC prognosis	MLP accuracy: 63%

We describe the prognostic models constructed by different AIs in different types of tumors and recount the methods they used, the study population, the characteristics and limitations, and the performance, and we focus on the models published in the last year

Currently, there are available radioactive substance analysis and CNN-based PET/CT image prognosis techniques. However, there are intrinsic restrictions to risk stratification when obtaining radiomics or deep features in grid Euclidean space. To accurately stratify HNC risk, a functional-structural subregion graph convolutional network (FSGCN) has been proposed [204]. To overcome challenges in predicting the LNM status from original cancer histology, Siteng Chen et al. [205] presented an attention-based weakly supervised neural network that relied on self-supervised cancer-invariant characteristics, which might function as an innovative prognostic marker across different types of cancers.

The combination of ML and DL is gaining increasing attention. Even after curative resection, pancreatic ductal adenocarcinoma (PDAC) has a dismal prognosis. The prognosis may be improved by using a DL-based classification of postoperative survival in the preoperative setting to guide treatment choices. Based on this, ensemble learning was used to merge two models that were separately constructed using clinical data-based ML models and computed tomography (CT) data-based DL models [206]. The classification of CRC tissues based on anatomical histopathological information, however, may not be possible using DL structures alone. In one study [207], data were input into a deep SVM based on an ensemble learning technique called DeepSVM after the features were chosen, and the results showed that the hybrid model had an accuracy of between 98.75 and 99.76% on CRC datasets.

## AI in clinical decision-making

The data required by physicians to make medical decisions are dispersed over numerous records, including a patient’s case history, test results, and imaging studies. Clinical prediction models usually use direct physician inputs or structured inputs taken from the electronic health record (EHR). The dependency on formatted inputs adds complexity to data processing as well as to the creation and use of models, resulting in the generation of many AI models. The invention of new drugs for oncology research in the era of precision medicine and the emergence of various treatment modalities, such as radiation therapy and surgery, have made the choice of oncology treatments fraught with various challenges. Given the breakthroughs in ML due to the availability of vast volumes of data, clinical decision-support systems (CDSS) driven by AI have been developed [229]. The earliest extensively used CDSS, Watson for Oncology (WFO, IBM Corporation, USA), has steadily gained popularity throughout the world in the treatment of thyroid carcinoma [230], prostate cancer [231], lung cancer [232], and breast cancer [233, 234]. Medical personnel enter a case’s structured data into the WFO system, and then, the most common treatment technique for the individual situation is quickly output by the system, along with reliable proof.

Beyond skilled medical professionals, AI algorithms can forecast certain medical outcomes for assisting clinical decision-making in many ways. In terms of digital data, transforming unstructured electronic health information into repomics (report omics) characteristics, a radiological repomics-driven model combining medical token cognition (RadioLOGIC) [235] is presented to evaluate human health and forecast pathological prognosis by transfer learning. The system exhibits superior feature extraction performance compared to cohort models and shows potential for automated clinical diagnosis verification from electronic health information. To predict outcomes and identify prognostic characteristics that correspond with both favorable and negative outcomes, the multimodal, poorly supervised deep learning system is able to integrate disparate modalities in whole-slide pictures and molecular profile data from 14 cancer types [236].

**Fig. 4:**
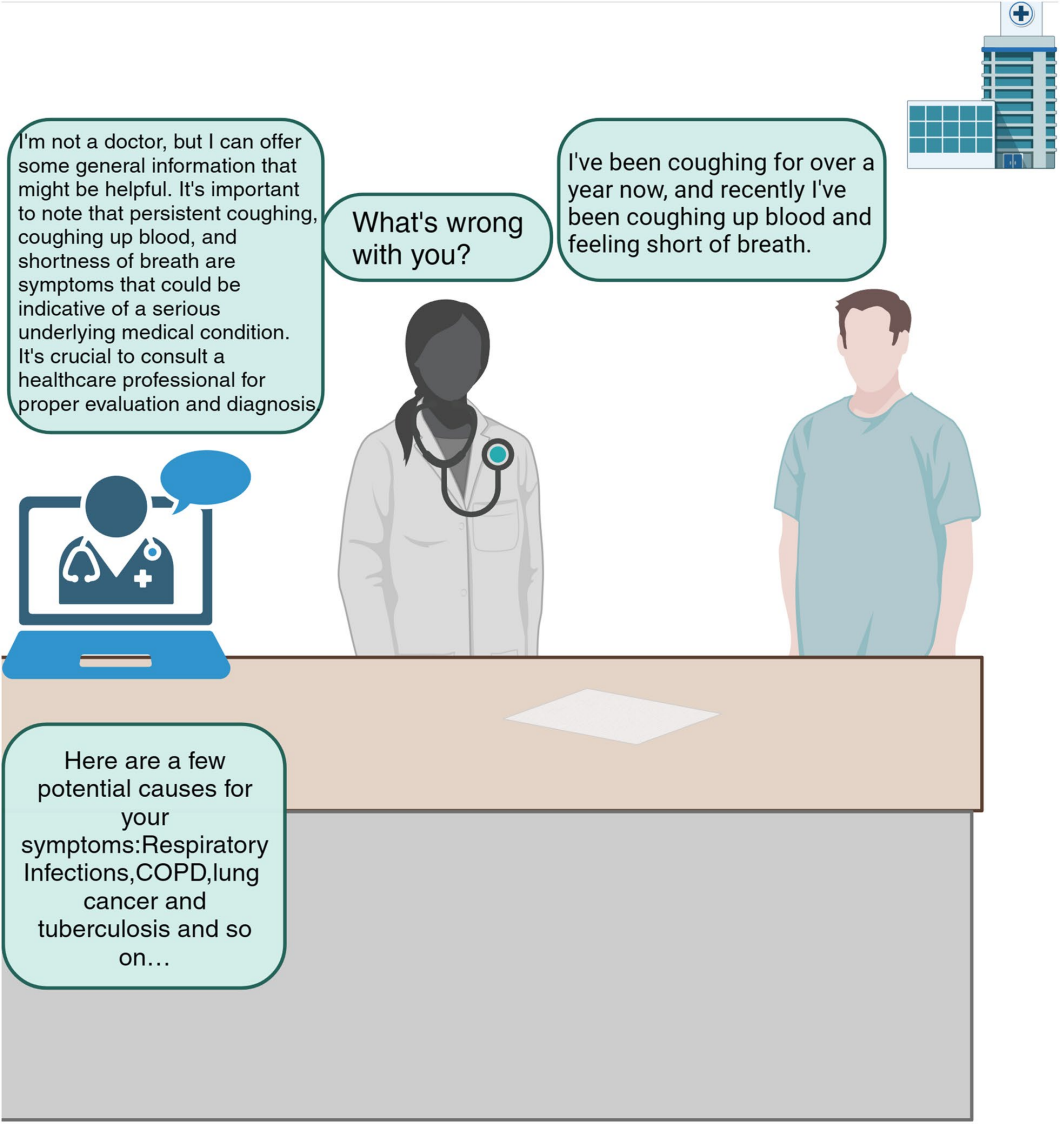
Simulates how a large language model (exemplified by ChatGPT) will assist doctors in diagnosing the disease after a patient with suspected lung cancer arrives at the hospital. Sending the chief complaint to ChatGPT, it would first emphasize that it is not a doctor itself, then warn that the symptoms are indicative of a serious illness, and give possible diseases. (Created with BioRender.com)

Although the CDSS can quickly collect and categorize stored information, the current state of application is that the CDSS is dominated by hospital ratings, and there is very little true large-scale application. EHR limitations, such as the inability to conduct efficient interpretation and information retrieval, can be addressed with the use of LLMs. LLMs are one of the most intriguing new advances in contemporary AI studies [237]. They receive training on billions of words taken from books, articles, and other online information. LLMs can perform data compression and encryption to protect data privacy. In cancer and medicine, DL natural language processing (NLP) with free-text analysis is being increasingly employed [238, 239]. Transformer models have taken over NLP [240]. To evaluate the accuracy of LLMs for deducing the cancer disease response from free-text radiology reports, a study compiled 10,602 computed tomography records from cancer patients examined at a single institution [241]. The results demonstrated that the GatorTron transformer, which had an accuracy of 0.8916 on the test set, outperformed bidirectional long- and short-term memory models, CNN models, and conventional ML techniques. This implies that transformer models may be employed as decision-support tools to offer doctors automatic second perspectives on illness responses. ChatGPT is the most representative LLM, and numerous cancer studies related to it have emerged since its introduction. It is substantially more accurate than previous large-scale language models when responding to queries concerning lung cancer [242], liver cancer [243], and prostate cancer [244]. Figure 4 indicates a response after sending a patient’s chief complaint to ChatGPT.

Another excellent example is Med-PaLM [245], a Google-developed chatbot for medical Q&A. Most evaluations of a model’s clinical expertise are automated and based on a small number of standards. MultiMedQA, a benchmark incorporating six existing medical question answering datasets, was introduced to solve these constraints. It surpasses the previous state of the art by more than 17% and has 67.6% accuracy on MedQA (questions similar to those on the US Medical Licensing Exam). However, the answers provided by the model compared to those provided by a physician still have a great deal of opportunity for improvement, as demonstrated by this work and similar studies. The follow-up Med-PaLM 2 scored 86.5% on the MedQA dataset, an improvement of more than 19% over Med-PaLM.

The development of LLMs completely equips cancer practitioners with tools that may be utilized to enhance the efficacy of therapy and the accuracy of tumor diagnosis as well as serve as a guide for clinical decision-making. In addition, regular individuals may utilize these platforms to identify particular clinical symptoms, which can aid in early identification and raise public awareness of health issues. Additionally, if people utilize AI, they may more quickly detect overmedication and improper treatment prescribed by some doctors in exchange for payment.

## Challenges and opportunities for the future

The future of AI in cancer research is fraught with both formidable obstacles and bright prospects for advancing cancer detection, diagnosis, therapy, and research.

### Availability and reliability of data

A significant quantity of training data is required for DL to be effective and credible. Limited data might lead to overfitting and a subpar performance in an external test cohort. Obtaining enough data is quite difficult when creating AI-based models, especially DL models. Data from medical imaging cannot be immediately entered. Processing and extracting information from the image data are essential. A typical neural network cannot fit all medical images, especially whole-slide images that can easily have billions of pixels per image. One method [246] is to crop the image before sending it to an AI system, adding a manual step to what may otherwise be a fully automated approach, to isolate a smaller region of interest, such as a portion of a slide image that contains a tumor. Insufficient labeling required for supervised learning can also lead to loss of data reliability. Additionally, issues occur when bias in datasets is caused by technical variables. Single-source bias, for instance, occurs when a single system generates an entire dataset. On the one hand, models can be trained on site-specific data to adapt to the unique characteristics of each location where they are used, and they are additionally developed and verified on datasets gathered from various sources to increase generalization [247]. On the other hand, biomedical technologies such as CODEX and spatial transcriptomics are one way to combine the picture and molecular data [136, 248]. These technologies overlay geographically resolved transcriptomics and proteomics data on images, enabling models to handle omics data in image form.

### Interpretability

Over the last 5 years, research into explainable AI has accelerated. DL has come under fire for being a “black box” that does not clarify the way the model transforms given inputs into outputs. It is challenging for oncologists to comprehend how DL models assess data and make judgments because of the numerous elements involved. The biological significance of explanatory ability must be thoroughly studied in order for DL to be approved by regulators and used as a diagnostic tool. In genomics, this requires comparing significant genetic traits found by DL to those identified by traditional bioinformatics techniques. Additionally, when a DL model is unsure about its predictions, its capacity to generate the “don’t know” output is crucial. Overconfidence in forecasts, such as forecasting the cancer main site with only 40% accuracy, can lead to erroneous cancer diagnosis or management decisions in crucial situations. Both post hoc and integrated interpretability approaches are viable ways to gather explanations from trained models and help the model learn to provide predictions and explanations concurrently.

### Ethics and morality

An increasing number of ethical questions around patient autonomy, prejudice, and transparency have been raised by the application of artificial intelligence (AI) in medicine [249]. The most susceptible source of information determines the total security level when we combine patient data from other sources. Clinical data are frequently the property of particular institutions due to concerns about patient privacy, and there are few methods in place to share data among institutions. It is frequently inadequate to remove personal identifiers and secret information since an attacker can still draw conclusions to retrieve some of the missing data. The good news is that multicenter information transfer agreements and safeguarding privacy distributed DL (DDL) are starting to overcome this roadblock [250–252]. DDL offers a mechanism that protects privacy so that several users can collaborate on learning using a deep model without directly exchanging local datasets. In addition, it is important to ascertain the level of supervision that doctors must provide and identify the person accountable for any poor choices made by DL tools. On the other hand, we should educate AI users to guarantee that they are knowledgeable consumers of the technology and endeavor to openly and clearly express to them what they should anticipate in a variety of circumstances. Many of the hazards described above may be reduced by being accessible, having varied demands, and being cautious.

When implementing AI, ethical issues are crucial since unethical data gathering or usage practices might introduce biases into models. These biases can take numerous forms, but they are mostly determined by the data and cohort composition employed by the particular AI systems. Providing and reviewing AI models lacks defined criteria or norms. Identifying the possible biases included in the established systems will be crucial; thus, future studies should fill this knowledge gap to help researchers and physicians.

### Clinical integration

As mentioned above, AI has been shown in many studies to improve the correctness of cancer diagnosis. However, a different perspective has been proposed. In one study [253], the authors systematically evaluated 131 published studies using the QUality Assessment of Diagnostic Accuracy Studies-2 (QUADAS-2) tool. They reported that the accuracy of AI in breast cancer screening programs cannot currently be evaluated based on available research, and it is unclear where in the therapeutic pathway AI could be most helpful.

For LLMs, one of their advantages is the capacity to sift through vast volumes of data and provide replies in a conversational and understandable manner. LLMs also have the potential to be used in patient education and consultation, offering patient-friendly information to aid in their understanding of their medical issues and available treatment choices, facilitating joint decision-making. More crucially, LLMs can contribute to the democratization of medical knowledge by allowing anybody, regardless of location or socioeconomic position, quick access to reliable medical information. However, special attention needs to be paid to the fact that current LLMs are not yet capable of fully replacing doctors, as they may contain errors or omit key points in the responses. Although ChatGPT-4.0 was more accurate than the other tools, neither ChatGPT nor Google Bard or the Bing or Google search engines provided 100% accurate answers to all queries [242]. The much-anticipated Med-PaLM, while promising, is evaluated by multiple choice questions; however, real life is not multiple choice, and different clinical symptoms and specificities in different patients make clinical diagnosis more complex. While AI, such as ChatGPT-4.0, might be helpful for giving broad information and responding to frequently asked queries. Nonetheless, it is important to take great caution when responding to inquiries from certain patients. It is essential to continuously upgrade AI models to include the most recent medical information.

Currently, almost all relevant AI models have been created to assist in cancer diagnosis using clinical data from the time of development. These clinical data may be derived from patient reports, complaints, or sequencing results. The question is whether there is an AI model that can recommend more tests and treatment modalities or perhaps aid in prescribing anticancer medication without relying on clinical data. The current state of affairs is that with the development of multiomics, a variety of data, such as methylation and fragmentomics [254], are being used to train AI models. If one day the data of the AI model accumulates to a large enough size, is it possible to predict the probability of cancer occurrence by only entering the data of normal people, and is it possible to give the corresponding chemotherapy regimen by only comparing the sequencing results of cancer patients and the database. This is a question worth thinking about and very interesting. First, the database must be large enough and ethical; second, there is variability between individuals, and it would be irresponsible to treat them by looking only at sequencing data at the genetic level or transcriptional level, for example.

However, if it is only in the area of cancer diagnosis, AI models have the potential to identify molecules and biomarkers associated with mutated genes and thus confirm the diagnosis of cancer independently of traditional pathology measurements. Meanwhile, with the advent of wearable and portable medical instruments, AI has shown much potential for the early screening of tumors. Therefore, we think that in the future, AI models have the potential to impact the cancer diagnostic market, but in terms of treatment, they cannot be separated from doctors and clinical data.

What must be realized is that despite the rapid development and promising future of AI, it can never replace clinicians and will only become an important tool to assist them in the future.

## Conclusion

In summary, AI has the ability to fundamentally alter cancer treatment and move it closer to the promise of precision oncology. In an era where genomics is being incorporated into health delivery and health data are being more digitized, it is anticipated that AI would be used in the construction, verification, and application of decision-support tools to promote precision oncology. We highlighted several promising AI applications in this review, including detection, prognosis, and administration of cancer treatments. It is undeniable that large language model can greatly assist physicians in their clinical work, but it can never replace them. Important conditions for the general adoption of AI in clinical settings include phenotypically rich data for the development of models and clinical validation of the biological value of AI-generated insights. Finally, clinical validation of AI is required before it may be used in ordinary patient treatment.

## Data Availability

Not applicable.

## Abbreviations

AI: Artificial Intelligence
AUC: Area under the curve
CC: Colon cancer
ccRCC: Clear cell renal cell carcinoma
CDSS: Clinical decision-support systems
cGANs: Conditional generative adversarial networks
CNNs: Convolutional neural networks
CRC: Colorectal cancer
CT: Computed tomography
DBT: Digital breast tomosynthesis
DDL: Distributed deep learning
DEGs: Differentially expressed genes
DEPs: Differentially expressed proteins
DFS: Disease free survival
DGCN: Directed Graph convolutional network
DL: Deep learning
DNNs: Deep neural networks
EHR: Electronic health record
GBM: Glioblastoma multiforme
GCN: Graph convolutional network
GPT: Generative pretrained transformers
H&E: Hematoxylin and eosin
HNC: Head and neck cancer
KIRC: Kidney renal clear cell carcinoma
KNN: K-Nearest neighbor
LDCT: Low-dose computed tomography
LLMS: Large language models
LNM: Lymph node metastasis
ML: Machine learning
MLSL: Multilabel softmax loss
MRI: Magnetic resonance imaging
MS: Mass spectrometry
MSI: Microsatellite instability
NCDs: Noncommunicable diseases
NLP: Natural language processing
NSCLC: Non-small cell lung cancer
OS: Overall survival
PET: Positron emission tomography
RSF: Random survival forest
ST: Spatial transcriptomics
SVM: Support vector machine
TCGA: The Cancer Genome Atlas
TME: Tumor microenvironment
TNM: Tumor node metastasis
USMLE: United States Medical Licensing Examination
WFO: Watson for Oncology
WSI: Whole-slide image
